# Mutual Information for Testing Gene-Environment Interaction

**DOI:** 10.1371/journal.pone.0004578

**Published:** 2009-02-24

**Authors:** Xuesen Wu, Li Jin, Momiao Xiong

**Affiliations:** 1 School of Life Science, Theoretic Systems Biology Laboratory and Center for Evolutionary Biology, Fudan University, Shanghai, China; 2 CAS-MPG Partner Institute of Computational Biology, SIBS, CAS, Shanghai, China; 3 Human Genetics Center, School of Public Health, The University of Texas Health Science Center at Houston, Houston, Texas, United States of America; 4 Department of Epidemiology and Statistics, Bengbu Medical College at Bengbu, Anhui, China; Peninsula Medical School, United Kingdom

## Abstract

Despite current enthusiasm for investigation of gene-gene interactions and gene-environment interactions, the essential issue of how to define and detect gene-environment interactions remains unresolved. In this report, we define gene-environment interactions as a stochastic dependence in the context of the effects of the genetic and environmental risk factors on the cause of phenotypic variation among individuals. We use mutual information that is widely used in communication and complex system analysis to measure gene-environment interactions. We investigate how gene-environment interactions generate the large difference in the information measure of gene-environment interactions between the general population and a diseased population, which motives us to develop mutual information-based statistics for testing gene-environment interactions. We validated the null distribution and calculated the type 1 error rates for the mutual information-based statistics to test gene-environment interactions using extensive simulation studies. We found that the new test statistics were more powerful than the traditional logistic regression under several disease models. Finally, in order to further evaluate the performance of our new method, we applied the mutual information-based statistics to three real examples. Our results showed that P-values for the mutual information-based statistics were much smaller than that obtained by other approaches including logistic regression models.

## Introduction

Complex diseases are the consequence of the interplay of genetic and environmental factors. Development of disease is a dynamic process of gene-gene and gene-environment interactions within a complex biological system which is organized into complicated interacting networks [1]. Modern complex theory assumes that the complexity is attributed to the interactions among the components of the system, therefore, interaction has been considered as a sensible measure of complexity of the biological systems. The more interactions between the components, the more complex system. We argue that the interactions hold a key for dissecting the genetic structure of complex diseases. Ignoring gene-environment interactions will likely mask the detection of a genetic effect and may lead to inconsistent association results across studies [2], [3].

Despite current enthusiasm for investigation of gene-environment interactions, published results that document these interactions in humans are limited, and the essential issue of how to define and detect gene-environment interactions remains unresolved. The concept of gene-environment interactions is often used, but rarely specified with precision [4]. Over the last three decades, epidemiologists have debated intensely about how to define and measure interaction in epidemiologic studies [5]. Many researchers indicated the importance of distinguishing biological interaction and statistical interaction [6]–[10]. Biological interaction between the gene and environment is often defined as the interdependent operation of genetic and environmental factors that cause diseases. In contrast, statistical interaction between the gene and environment is defined as the interdependence between the effects of genetic and environmental risk factors in the context of a statistical model. The effects of disease risk factors are often measured by relative risks and odds ratios. The classical definition of statistical interaction has the following limitations. First, both relative risks and odds ratios are mainly defined for binary variables. Their extensions to multiple categorical risk factors (for example, three genotypes and multiple categorical environments) are cumbersome. Second, statistical interactions are essentially model dependent. Linear models and generalized linear models (logistic regressions and log-linear models) of the genetic effects of the risk factors are often used to define statistical interactions. In the classical logistic regressions and log-linear models of the gene-environment interactions, the genetic effects of the risk factors are decomposed into main effects and interaction effects (or product term) in the model. But, such decomposition may not reflect the true nonlinear interaction between the gene and environment. In addition, in these models, the major part of the true biological interactions between the gene and environment is often partitioned into the marginal effects. The remaining part of the gene-environment interactions which is treated as a departure (or residual) from the logistic regression and log-lineal models is small and hard to detect. Third, the classical interaction models can hardly be applied to study interactions (including pair-wise and high-order interactions) among the components of the biological systems and their complexity.

To overcome the limitations of the classical definition of the statistical interaction, we propose a new definition of interaction that is based on interdependence among the risk factors causing disease. Interactions between genes and the environment can generally be defined as a stochastic dependence between genetic and environmental risk factors in causing phenotypic variation among individuals. This definition does not require specifying the statistical models of the risks, and is similar, although not exactly identical, to the definition of biological interaction. The concept of mutual information proposed by Shannon [11] can serve as a general measure of interaction (dependence) between two random variables [12]–[14]. An additional asset is that mutual information measures more than linear dependence [15]–[16]. As we will show in the methods section, mutual information between the gene and environment has a close relationship with the classical measures of the gene-environment interactions such as odds ratio and relative risk.

As Liu [17] pointed out, “the success of investigation of G×E interactions depends greatly on the selection of the optimal study design, the most accurate and precise assessment of genetic and environmental factors, and the most efficient statistical analysis”. Developing efficient analytic methods for evaluation of the gene-environment interactions is critical to the investigation of gene-environment interactions [18].

Odds ratio calculations, logistic regression analysis, data mining and multifactor dimensionality reduction (MDR) are some of the existing methods available to evaluate the gene-environment interactions [19]–[30]. These methods have their merits, but also they have limitations. As an alternative to these widely used methods for testing gene and environment interactions, we propose mutual information-based methods to detect gene and environment interactions.

The main purpose of this report is to use information theory as a general framework for developing statistical methods to detect gene-environment interactions. To accomplish this, we first developed a novel definition of gene-environment interactions. Then we studied how to use mutual information to measure gene-environment interactions. We investigated how gene-environment interactions generate the large difference in aforementioned measures between the general population and disease population. This provided the motivation to develop mutual information-based statistics for testing gene-environment interactions. Using extensive simulation studies, we validated the null distribution and type 1 error rates of the mutual information-based statistics for testing gene-environment interactions. To reveal the merit and limitation of the mutual information-based statistics to detect gene-environment interactions, we compared their power for detecting gene-environment interactions with the logistic regression. We found that the new test statistics have higher power than the traditional logistic regression under several disease models. Finally, in order to further evaluate the performance of our new method, we applied the mutual information-based statistics to real data examples. Our results showed that P-values for the mutual information-based statistics were smaller than that obtained by other approaches including logistic regression models.

## Methods

### Information measure of the gene-environment interaction

Consider a disease susceptibility locus G and an environment E. The locus G has three genotypes coded as 0, 1, and 2. The environmental exposure is coded as E = 1, otherwise E is coded as 0. Let D be an indicator of disease. Mutual information measures dependence between two random variables. The mutual information between the gene and environment in the general population is defined as
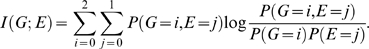
(1)Information theory [11] shows that mutual information 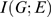 is equal to zero if and only if
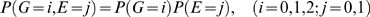
i.e., gene and environment variables are independent.

The mutual information between the gene and environment in the disease population is given by
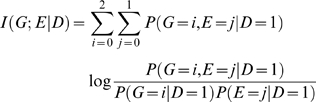
(2)while Equation (2) can be reduced to
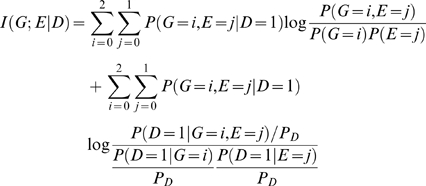
(3)where 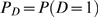 is the prevalence of the disease.

Equation (3) shows that mutual information 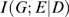 has two components. The first term in equation (3) is due to the dependence between the gene and environment in the general population. The second term in equation (3) is due to interaction. Thus, we define information measure of the interaction between the gene and environment as
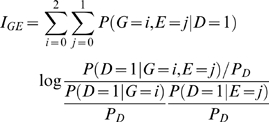
(4)which implies that 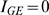 if and only if
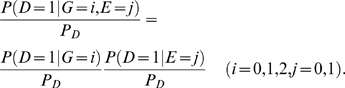
(5)


Information measure of interaction has two remarkable features. First, it is defined in terms of penetrance and hence related to the cause of the disease. Second, the interaction is measured by the interdependent operation of the gene and environment in causing disease. Absence of the gene and environment interaction indicates that equation (5) should hold.

If we assume that the gene and environment variables in the general population are independent, then
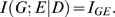
In this case, the mutual information between the gene and environment in the disease population is equal to the information measure of the interaction between gene and environment. This provides an easy way to calculate the information measure of gene-environment interactions.

To gain understanding of the information measure of the gene and environment interaction, we studied several special cases.

**Case 1:** G is not the disease locus. If we assume that G is only a marker and will not cause disease, then we have
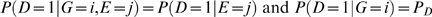
which implies that
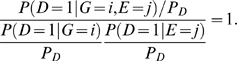
Thus, we obtain 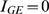. In other words, if the locus G is a marker, there is no interaction between the locus G and environment. The interaction measure 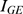 between the marker and environment should be equal to zero. Hence, our information measure of the gene-environment interactions correctly characterizes the marker case.

**Case 2:** Environmental exposure will not cause disease. If the environmental exposure will not cause disease, there will be no interaction between the gene and environment. We expect that the information measure of gene and environment interaction should be equal to zero. Indeed, by the same argument as provided in case 1, we can show this.

### Test statistics

In the previous section, we show that the information measure of the gene-environment interactions is related to the dependency of the gene and environment variables in the disease population. The interaction can be detected by testing the independence of the gene and environment. Before defining the test statistic, we introduce the following notations. Let
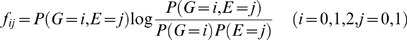
and 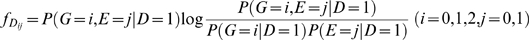. Let 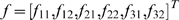 and 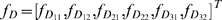 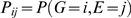 and 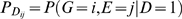. Define
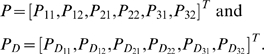
 The joint probabilities of the gene and environment variables in both the general population and disease population follow multinomial distributions with the following covariance matrices.
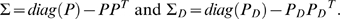
Let the Jacobean matrices of 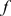 and 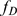 with respect to 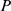 and 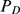 be 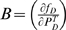 and 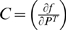, respectively. It is easy to see that
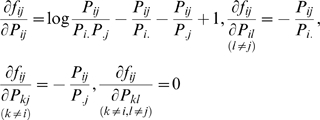
where 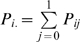, and 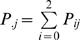. The partial derivatives of the function 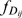 with respect to 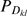 can be similarly defined. Let 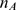 be the number of sampled individuals in the cases and 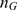 be the number of sampled individuals in the controls. Define
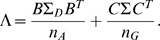
The statistic for testing the gene-environment interactions is then defined as
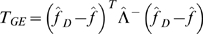
(12)where 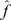, 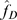, and 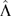 are the estimators of 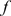, 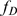, and 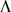. 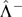 is a generalized inverse of the matrix 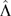


When the sample size is sufficiently large enough to ensure application of the large sample theory, the test statistic 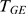 is asymptotically distributed as a central 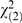 distribution under the null hypothesis of the no gene-environment interactions, if we assume that the gene and environment variables in the general population are independent (Appendix S1).

We can also develop a statistic for testing interaction between each genotype and environment. For example, for genotype 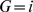, let
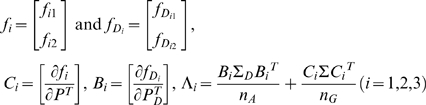
then, the statistic for testing interaction between the genotype 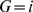 and environment is defined as
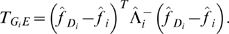
(13)Under the null hypothesis of no interaction between the genotype 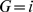 and the environment the statistic 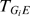 is asymptotically distributed as a central 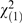 distribution.

## Results

### Null distribution of test statistics

**Figure 1:**
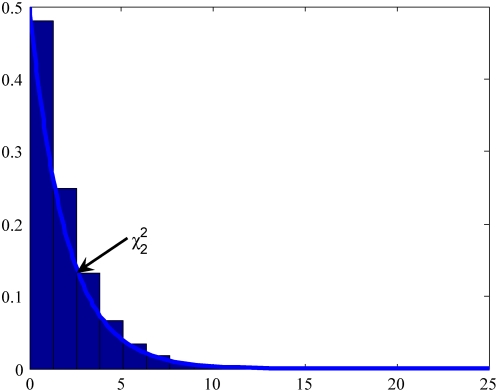
Null Distribution of the statistic 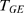 for 400 cases and controls respectively.

In the previous section we stated that the test statistic 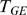 and 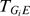 under the null hypothesis are asymptotically distributed as a central 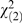 distribution and a central 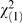 distribution, respectively, if we assume that the gene and environment variables are independent in the general population. To validate this statement we performed a series of simulation studies. The computer program SNaP [31] was used to generate the genotype data of the individuals and MATLAB was used to randomly generate the environment data of the individuals. Individuals (n = 100,000) with independent genotype and environmental exposure where the frequencies of two alleles at the locus were equal, and the frequency of the environmental exposure was equal to 0.2 (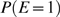) were generated and equally divided into cases and controls. A total of 20,000 simulations were repeated. We plot Figures 1–4 showing the histograms of the test statistics 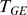 and 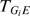 for testing the interaction between the gene and environment, with sample sizes 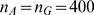, where 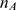 and 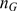 are the number of sampled individuals in the cases and controls. Figures 1–4 show that the null distributions of the test statistics 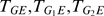 and 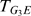 are similar to the theoretical central 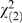 and 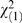 distributions, respectively.

**Figure 2 pone-0004578-g002:**
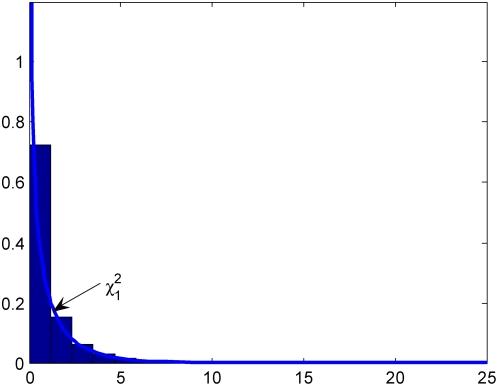
Null Distribution of the statistic 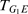 for 400 cases and controls respectively.

**Figure 3 pone-0004578-g003:**
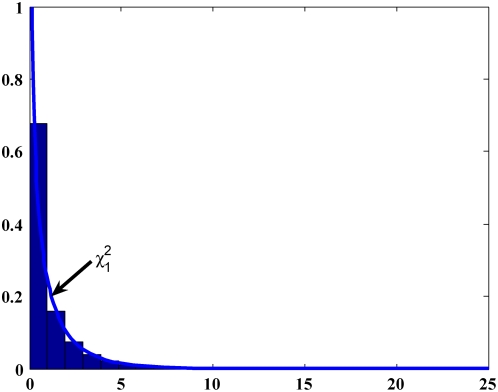
Null Distribution of the statistic 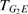 for 400 cases and controls respectively.

**Figure 4:**
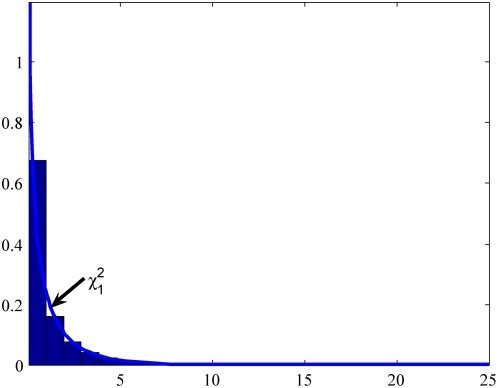
Null Distribution of the statistic 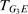 for 400 cases and controls respectively.

Type I error rates were calculated by random sampling 200–1,000 individuals from each of the cases and controls. In Tables 1 and 2 we listed type I error rates for 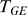 and 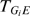, assuming 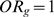 and 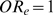. In Table 3 we listed type I error rates for 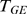, assuming 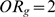 and 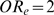 (For 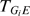, in case of 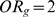 and 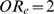 we can obtain the similar results (data not shown). Tables 1–3 demonstrated that the estimated Type I error rates for the statistics 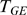 and 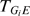 to test the gene and environment interactions were not appreciably different from the nominal levels 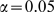, 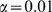 and 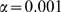, which were independent of the gene and environment odds ratios 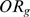 and 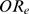.

**Table 1:** Type 1 error rates for the test statistic 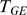 to test gene-environment interaction, assuming 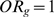 and 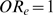.

Sample size	Nominal levels		
	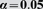	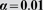	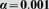
200	0.04641	0.00845	0.00076
300	0.04618	0.00871	0.00089
400	0.05033	0.00964	0.00098
500	0.04811	0.00902	0.00082
600	0.05012	0.01002	0.00082
700	0.04991	0.00948	0.00096
800	0.04804	0.00953	0.00098
900	0.04737	0.00840	0.00088
1000	0.04926	0.00979	0.00107

**Table 2 pone-0004578-t002:** Type 1 error rates for the test statistic 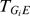 to test gene-environment interaction, assuming 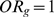 and 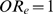.

Sample size	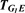			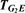			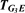		
	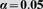	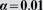	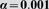	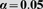	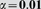	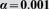	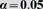	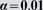	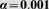
200	0.0495	0.0097	0.0010	0.0507	0.0104	0.0013	0.0486	0.0093	0.0009
300	0.0482	0.0094	0.0008	0.0503	0.0100	0.0010	0.0473	0.0094	0.0009
400	0.0494	0.0092	0.0009	0.0490	0.0103	0.0011	0.0493	0.0098	0.0010
500	0.0491	0.0089	0.0008	0.0493	0.0105	0.0014	0.0479	0.0095	0.0010
600	0.0480	0.0096	0.0010	0.0498	0.0098	0.0011	0.0494	0.0096	0.0010
700	0.0500	0.0100	0.0012	0.0492	0.0095	0.0008	0.0484	0.0102	0.0009
800	0.0494	0.0097	0.0011	0.0494	0.0096	0.0010	0.0474	0.0090	0.0008
900	0.0489	0.0092	0.0008	0.0497	0.0103	0.0013	0.0494	0.0095	0.0009
1000	0.0482	0.0095	0.0009	0.0506	0.0108	0.0013	0.0488	0.0090	0.0007

**Table 3:** Type 1 error rates for the test statistic 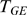 to test gene-environment interaction, assuming 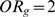 and 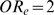.

Sample size	Nominal levels		
	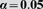	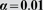	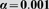
300	0.0513	0.0102	0.0012
400	0.0473	0.0096	0.0007
500	0.0470	0.0087	0.0011
600	0.0482	0.0100	0.0008
700	0.0513	0.0102	0.0015
800	0.0479	0.0100	0.0011
900	0.0493	0.0089	0.0005
1000	0.0494	0.0102	0.0010

### Power evaluation

To evaluate the performance of the mutual information-based statistic for testing gene-environment interactions, we compared its power to that of the logistic model. The computer program SNaP [31] was used to generate the genotype data of the sampled individuals and MATLAB was used to randomly generate the environmental data of the sampled individuals. A population of 500,000 individuals with independent genotype and environmental exposure where the minor allele frequency (MAF) at the locus were equal to 0.3 and the frequency of the environmental exposure was equal to 0.2 (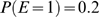) was generated. The model of the disease with the gene and environment interaction was defined by the penetrance. Gene-environment interactions effects were simulated with penetrance functions as given in Appendix S2. We assume the prevalence of the disease 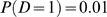.

We consider two cases: (1) genetic and environmental odds ratios: 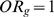 and 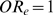, and (2) 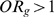 and 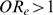. In case (1), definition of the absence of the gene-environment interactions by information measure and gene-environment odds ratio in the logistic regression model is equivalent. In case (2), the information measure of interaction covers more situations which are interacted under the definition of information measure, but not interacted under definition of logistic models.

**Figure 5:**
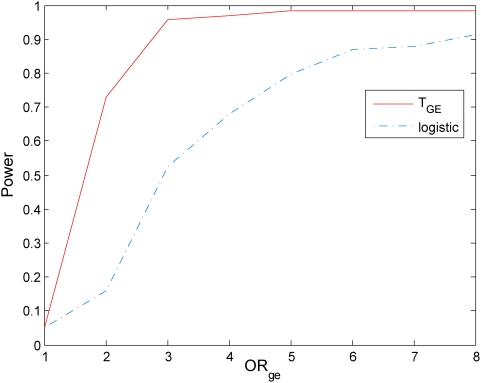
Power of the statistic *T_I_* and logistic regression analysis for 300 cases and controls respectively.

With this disease model, we randomly generated a disease population with 10,000 affected individuals and a general population with 10,000 unaffected individuals from the population of 500,000 individuals. We then randomly sampled 500 individuals (cases) from the disease population and 500 individuals (controls) from the general population. We repeated 20,000 simulations. We presented six panels of Figures to compare the power of the proposed mutual information-based statistic and logistic regression models. Power calculation of logistic regression is based on the model 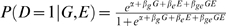. Figures 5–7 plot the power of the test statistic 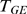 and logistic regression to detect the gene-environment interactions in case (1) (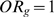 and 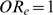) as a function of the gene-environment interactions odds ratios under the significance level 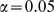 for sample sizes 300, 400 and 500, respectively. Figures 8–10 plot the power of the test statistic 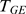 and logistic regression to detect the gene-environment interactions in case (2) (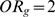 and 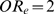) as a function of the gene-environment interactions odds ratios under the significance level 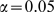 for sample sizes 300, 400 and 500, respectively. These figures showed that the power of the mutual information-based statistic is much higher than that of the logistic regression even if in case (1) where the definition of absence of the gene-environment interactions by both the information measure and odds ratio in the logistic regression is equivalent. We also find that the difference in the power between the mutual information-based statistic and the logistic regression model became larger as the significance level increases (data are not shown).

**Figure 6 pone-0004578-g006:**
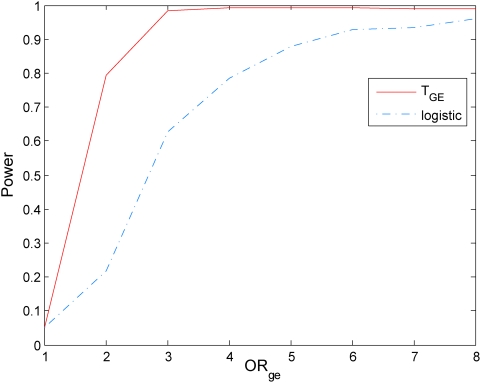
Power of the statistic *T_I_* and logistic regression analysis for 400 cases and controls respectively.

**Figure 7:**
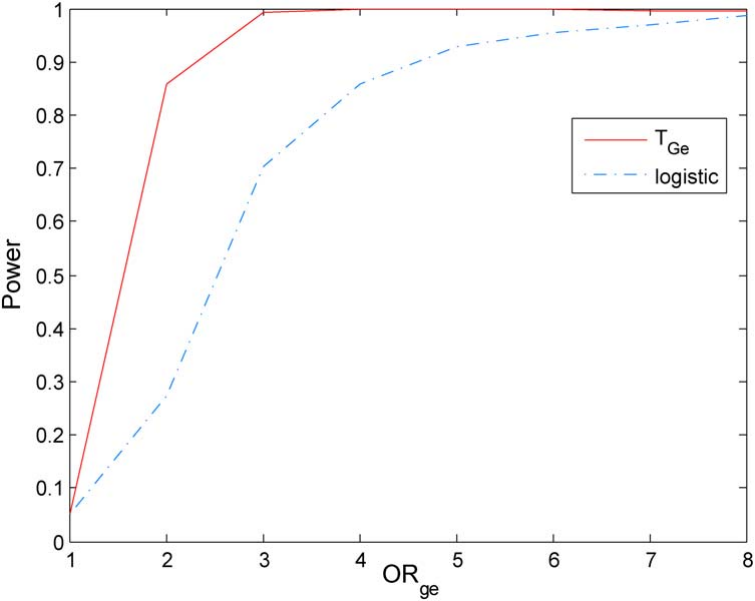
Power of the statistic *T_I_* and logistic regression analysis for 500 cases and controls respectively.

**Figure 8:**
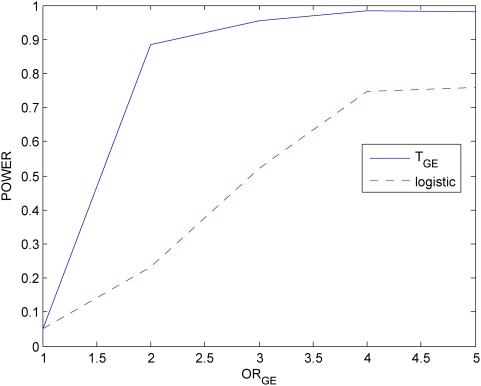
Power of the statistic *T_I_* and logistic regression analysis for sample size 300, 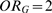 and 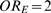.

**Figure 9 pone-0004578-g009:**
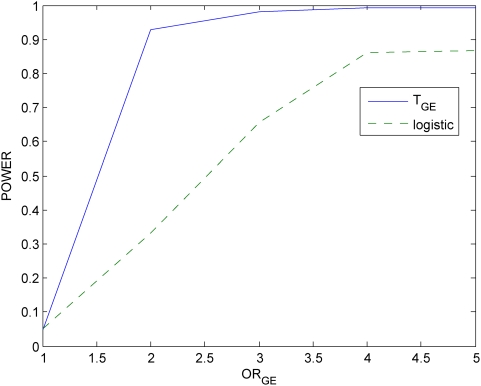
Power of the statistic *T_I_* and logistic regression analysis for sample size 400, 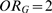 and 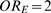.

**Figure 10:**
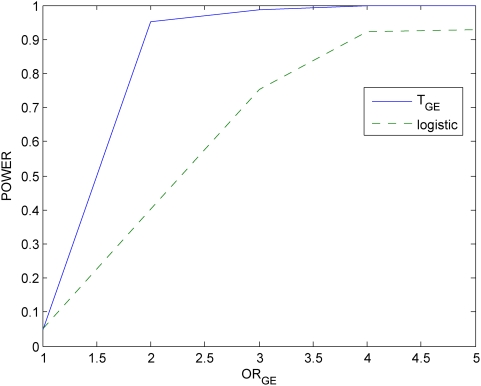
Power of the statistic *T_I_* and logistic regression analysis for sample size 500, 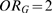 and 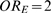.

### Application to real data example

To further evaluate its performance for testing gene-environment interactions, the mutual information-based statistics 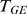 and 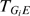 were applied to real data examples. The first example studied the interaction between the polymorphism of the gene excision repair cross-complementing group 2 (ERCC2) and smoking exposure in lung cancer [32], where two ERCC2 polymorphisms Asp312Asn and Lys751Gln were typed in 1,092 Caucasian lung cancer patients and 1,240 spouse and friend controls collected at Massachusetts General Hospital. Both ERCC2 polymorphisms in the controls were in Hardy-Weinberg equilibrium. Smoking exposure was classified into four categories: non smoking, mild smoking, moderate smoking and heavy smoking. For simplicity of comparison, we performed only crude analysis. In other words, analysis was performed only for the raw data that were not adjusted for age and gender. Both the mutual information-based statistics and logistic regression analysis were used to test interaction between the polymorphism of ERCC2 and smoking in lung cancer. The results were summarized in Table 4. In general, the logistic regression will not be used to test interaction between a single genotype and environment, thus there was no p-value to test interaction between the single genotype and environment for logistic regression in Table 4. Two features emerge from Table 4. First, in general, the p-values of the global test statistic 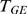 were smaller than that of the 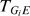 for testing interaction between the particular genotype (single genotype) and environment. Second, in most cases, the p-values of the mutual information-based global test statistic 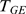 were smaller than that of the logistic regression analysis. ERCC2 is a major DNA repair gene. DNA repair genes play a key role in protecting the genome from damage caused by smoking [32].

**Table 4:** Comparison of p-values for the mutual information-based statistics and logistic regression to the interaction between ERCC2 polymorphisms and smoking in lung cancer.

Genotype	Smoking					
	Mild		Moderate		Heavy	
	P-values		P-values		P-values	
	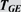 or 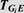	Logistic regression	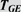 or 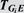	Logistic regression	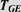 or 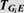	Logistic regression
Asp312Asn	0.0028	0.0151	5.70 E-4	0.0114	<10^−10^	2.53E-05
Asp/Asp	0.0679		0.3447		0.00051	
Asp/Asn	0.1116		0.2462		0.4735	
Asn/Asn	0.0082		0.0094		0.0036	
Lys751Gln	0.0535	0.1611	1.24E-08	5.24E-01	<10^−10^	0.00197
Lys/Lys	0.2010		0.0872		0.00095	
Lys/Gln	0.6391		0.3875		0.2417	
Gln/Gln	0.2364		0.4702		0.0399	

The second example is to study the interaction between the gene SULT1A1 and smoking/alcohol consumption for squamous cell carcinoma of the oesophagus [33]. The gene SULT1A1 catalyses sulfation that is related to the metabolism of a broad range of compounds such as phenolic xenobiotics, hydroxylated aromatic amines and drugs. The gene SULT1A1 is suspected to play a role in oesophageal cancer (OC). We applied the developed mutual information-based statistics and logistic regression to this dataset to test for gene-environment interactions in causing OC. The data in Table 5 were from Dandara's Table 4 [33] for the Mixed Ancestry South African group. The P-values in Dandara's Table 4 were obtained by the statistic based on odds ratios which tested for both the gene and environment interaction effects and the genetic effect. Thus, instead of using the P-values provided by Dandara, we used logistic regression to recalculate the P-values to test for interaction between the gene SULT1A1 and smoking or/and alcohol consumption using data from Table 4 in Dandara [33]. The P-values of both mutual information-based statistics and logistic regressions were listed in Table 5. We can see that using the mutual information-based statistics we detected the interaction between the gene SULT1A1 and smoking, or the combination of smoking and alcohol consumption in causing OC in the Mixed Ancestry South African group, however logistic regression analysis failed to make a similar detection. The mutual information-based statistic also needs much less time than logistic regression analysis. For this example, if we use Intel Pentium(R) (D CPU 2.66 GHz×2.66 GHz, 2G memory, Windows XP) the computation time for the mutual information-based statistic and logistic regression analysis was about 2.81×10^−4^ seconds and 1.59×10^−2^ seconds, respectively.

**Table 5:** Comparison of p-values for the mutual information-based statistics and logistic regression to the interaction between the gene SULT1A1 and smoking (alcohol consumption) in the Mixed Ancestry South African group.

SULT1A1 genotype	Patients		Controls		P-values	
					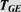 or 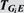	Logistic regression
Tobacco smoking						
	no	yes	no	yes	0.0194	0.5196
SULT1A1*1/*1	3	45	15	37	0.0536	
SULT1A1*1/*2	1	16	2	27	0.0096	
SULT1A1*2/*2	2	27	3	10	0.9152	
Alcohol consumption						
	no	yes	no	yes	0.0863	0.1847
SULT1A1*1/*1	12	36	31	21	0.2443	
SULT1A1*1/*2	2	15	13	16	0.4105	
SULT1A1*2/*2	9	20	5	8	0.1648	
Both smoking and alcohol consumption						
	no	yes	no	yes	0.0017	0.1902
SULT1A1*1/*1	2	35	15	21	0.0082	
SULT1A1*1/*2	1	15	2	16	0.0124	
SULT1A1*2/*2	2	20	2	7	0.4310	

The third example is case-control study of interaction between smoking and HLA-DR SE (shared epitope) gene in the development of anticitrulline antibody-positive rheumatoid arthritis (RA) in the Swedish Epidemiological Investigation of Rheumatoid Arthritis (EIRA) study [34], [35]. The major environmental risk factor and genetic risk factor are smoking and HLA-DA shared epitope (SE) gene, respectively. We analyzed data from Klareskog [34] which consisted of 827 RA patients and 1216 controls and from Kallberg [35] which consisted of 1883 RA patients and 1589 controls. Clearly, the second study [35] is the extension of the first study [34]. Both the mutual information-based statistic and logistic regression were applied to the dataset to test for interaction between the smoking and HLA-DR SE genes in the development of anticitrulline antibody-positive RA. The results were summarized in Table 6. They confirmed the recently pronounced interaction between smoking and the HLA-DR SE gene in the development of RA[34], [36]–[37]. We also see that as the sample size increased in the second dataset, the P-values became much smaller (from 0.000925 to <10^−10^). The results also again showed that the P-values of the mutual information-based statistics are usually smaller than that of the logistic regression and that the P-values of the global test statistic 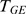 using all information at the locus are in general smaller than that of the test statistic 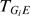.

**Table 6:** Comparison of p-values for the mutual information-based statistics and logistic regression to the interaction between smoking and HLA-DR SE genes in the development of anticitrulline antibody-positive RA.

Sex, anti-CCP status and HLA-DR SE genes	Case		Control		P-values	
	Never smoked	Ever smoked	Never smoked	Ever smoked	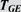 or 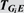	Logistic regression
The data were from Klareskog [34]						
Male and Female						
Anti-CCP^+^					9.25E-04	0.0198
No SE	20	58	87	184	0.01490	
Single SE	72	192	104	146	0.7090	
Double SE	36	126	31	31	0.03250	
Anti-CCP^−^					0.2245	0.1989
No SE	65	84	87	184	0.2037	
Single SE	64	76	104	146	0.4170	
Double SE	18	18	31	31	0.4585	
Female						
Anti-CCP^+^					0.2180	0.1378
No SE	18	41	74	115	0.4437	
Single SE	58	130	75	109	0.4989	
Double SE	30	89	25	25	0.08492	
Anti-CCP^−^					0.3128	0.8859
No SE	50	62	74	115	0.4577	
Single SE	45	52	75	109	0.9805	
Double SE	15	11	25	25	0.8092	
Male						
Anti-CCP^+^					6.72E-10	0.1172
No SE	2	17	13	69	2.55E-09	
Single SE	14	63	29	37	0.0574	
Double SE	6	37	6	6	0.2240	
Anti-CCP^−^					0.0273	0.01472
No SE	15	24	13	69	0.0244	
Single SE	19	24	29	37	0.1519	
Double SE	3	7	6	6	0.1423	
Date were from Kallberg [35]						
Anti-CCP^+^					<10^−10^	0.0059
No SE	35	71	137	242	0.0240	
Single SE	105	270	138	198	0.6392	
Double SE	61	179	39	39	0.0455	
Anti-CCP^−^					0.3844	0.2979
No SE	86	115	140	242	0.2795	
Single SE	87	123	138	198	0.3946	
Double SE	25	26	39	39	0.6170	

## Discussion

Over the last three decades, epidemiologists have debated intensely about how to define and measure interaction in epidemiologic studies [5]. The distinction between biological interaction and statistical interaction becomes an important issue [6], [39]. Biological interaction is often defined as interdependent operation of genetic and environmental factors that cause diseases. In other words, biological interaction means that joint presence of the genetic and environmental factors is the necessary condition for causing disease.

Due to the complexity of the development of the diseases, as Rothman [38] pointed out, there is no way to directly observe biological interactions. Biological interactions are often indirectly inferred. Our aim is to estimate the magnitude of the biological interaction as accurate as possible and develop efficient statistics to detect biological interactions. The purpose of this report is to address several issues for achieving this goal.

The first issue is how to define biological interaction mathematically. The major challenge is to come up with a definition that is mathematically explicit. In this report, we chose to use the classical concept of conditional probability to define biological interaction. A key component to biological interaction is the dependence of developing disease with the presence of both genetic and environmental factors. Therefore, the conditional dependence of the genetic factor on the environmental factor in causing disease is a natural expression for biological interaction. This mathematical definition is an alternative to the definition of biological interaction as a departure from additivity [38], [39]. With this definition, interaction has a broader meaning and divergent statistical and computational tools available for further analysis.

The second issue we addressed is how to measure gene-environment interactions. Mutual information is widely used in communication systems and complex adaptive systems analysis as a general measure of stochastic dependence between two random variables. In this report, we extended mutual information to measure gene-environment interactions. Widely used measures of interaction include relative risks or odds ratios which were originally defined for binary data. As a consequence, we often code genetic and environmental factors as binary variables for calculation of relative risks and odds ratios even if the genetic and environmental factors take multiple values or even continuous values. Mutual information can be defined for genetic and environmental factors with multiple values (or even continuous values, but not discussed here). Therefore, mutual information can cover broader cases than the relative risks and odds ratios.

The third issue addressed how to develop efficient statistics to detect gene-environment interactions. Despite current enthusiasm for investigation of interactions between the gene and environment, the essential issue of how to detect gene-environment interactions remains unresolved. Developing efficient analytical methods for evaluation of the gene-environment interactions is central to the investigation of gene-environment interactions [18]. Logistic regression is predominantly used to test for gene-environment interactions in epidemiology [38]. It depends on how to decompose the genetic effect. Most researchers use logistic regressions to model odds as the additive combination of main effects of a single-locus and the environment, and a residual term. The residual term in the model is defined as a statistical interaction between the gene and environment. As a consequence, the major part of functional (or biological) gene-environment interactions may be included in the main effects. The remaining part of the functional gene-environment interactions which is treated as a residual term in the mathematical model is small and hard to detect.

In this report, we presented mutual information-based statistics to detect gene-environment interactions. Through extensive simulation studies, we showed that the null distribution of the mutual information-based statistics was close to a central 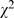 distribution. We also calculated type 1 error rates of the mutual information-based statistic by simulation. Our results showed that type 1 error rates were close to the nominal significance levels. We also investigated the power of the new statistic to detect the gene-environment interactions by analytical methods. It showed that the mutual information-based test statistics have a much higher power in detecting the interaction than logistic regression methods even when 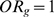 and 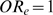 where definition of absence of interaction by both the information measure and odds ratio measure in the logistic regression are equivalent. To further evaluate their performance to detect the gene-environment interactions, the proposed mutual information-based statistics were applied to three published data sets. Our results showed that, in many cases, P-values of the mutual information-based statistics were much smaller than the results of the logistic regression analysis.

Since the computation time for the mutual information-based statistic is small, it is feasible to perform the genome-wide gene-environment interaction analysis using PC machines. As we reported in the previous section that the computation time for the mutual information-based statistic to test one interaction between the gene and environment (94 cases and 94 controls) was only 2.81×10^−4^ sec, the total time required for testing the gene-envronment interaction for 1,000.000 SNPs and thousands of cases and controls will be about one hour.

Although the preliminary results are appealing, the mutual information-based statistics for detection of gene-environment interactions also suffer from several limitations. First, they require an assumption that the genetic and environmental variables in the general population are independent. Deviation from independent assumption will affect the false positive rates. Second, they need to calculate the generalized inverse of the singular covariance matrix, which may lead to instability of numerical calculations. Third, in this report, we only compared the power of the mutual information-based statistic with that of the logistic regression. A comparison with other methods including methods based on defining interaction as a departure from additive effects is in progress.

Gene-environment interactions are an important, but complex concept. There are a number of ways to define gene-environment interactions. How the definition of gene-environment interactions in population level reflects their biochemical or physiological interaction is still a mystery. We hope that this work provides further motivation to conduct theoretical research and large-scale data analysis in deciphering the genetic and physiological meaning of gene-environment interactions and to develop more statistical methods for testing gene-environment interactions. In the coming years, to integrate gene-environment interactions into genome-wide association analysis will be a major task in genetic studies of complex diseases.

## Supporting Information

Appendix S1 (0.11 MB DOC)

Appendix S2 (0.05 MB DOC)
