# Supplementary material for: Mutual Information for Testing Gene-Environment Interaction
Source: PLoS One. 2009 Feb 24;4(2):e4578. doi: 10.1371/journal.pone.0004578 (PMC2642626; doi:10.1371/journal.pone.0004578)
Supplement: Appendix S1 — (0.11 MB DOC) [file pone.0004578.s001.doc]

**APPENDIX S1**

Let $C_{i}=[\frac{\partial f_{i}}{\partial P^{T}}]$, we first show that under the null hypothesis of no gene-environment interaction, the vector $C_{1}$ and $C_{2}$, $C_{3}$ and $C_{4}$,$C_{5}$ and $C_{6}$ satisfy

$|\left[\begin{matrix} C_{1} \\ C_{2} \end{matrix}\right]\Sigma\left[\begin{matrix} C_{1}^{T} & C_{2}^{T} \end{matrix}\right]|=0$, $|\left[\begin{matrix} C_{3} \\ C_{4} \end{matrix}\right]\Sigma\left[\begin{matrix} C_{3}^{T} & C_{4}^{T} \end{matrix}\right]|=0$ and $|\left[\begin{matrix} C_{5} \\ C_{6} \end{matrix}\right]\Sigma\left[\begin{matrix} C_{5}^{T} & C_{6}^{T} \end{matrix}\right]|=0$.

Recall that under the null hypothesis of no gene-environment interaction, we have
$P_{ij}=P_{i.}P_{.j}\begin{matrix} & (i=0,1,2;j=0,1) \end{matrix}$

Thus,

$C_{1}=[\begin{matrix} 1-P_{1.}-P_{.1}, & -P_{.1}, & -P_{1.}, & 0, & -P_{1.}, & 0 \end{matrix}]$ and $C_{2}=[\begin{matrix} -P_{.2}, & 1-P_{1.}-P_{.2}, & 0, & -P_{1.}, & 0, & -P_{1.} \end{matrix}]$

After some calculations, we obtain

$$\begin{matrix} \left[\begin{matrix} C_{1} \\ C_{2} \end{matrix}\right]\Sigma\left[\begin{matrix} C_{1}^{T} & C_{2}^{T} \end{matrix}\right]=\left[\begin{matrix} C_{1} \\ C_{2} \end{matrix}\right]diag(P)\left[\begin{matrix} C_{1}^{T} & C_{2}^{T} \end{matrix}\right]-\left[\begin{matrix} C_{1} \\ C_{2} \end{matrix}\right]PP^{T}\left[\begin{matrix} C_{1}^{T} & C_{2}^{T} \end{matrix}\right] \\ =\left[\begin{matrix} P_{11}(1-P_{1.}-P_{.1})^{2}+P_{12}P_{.1}^{2}+P_{21}P_{1.}^{2}+P_{31}P_{1.}^{2}, & -P_{11}P_{.2}(1-P_{1.}-P_{.1})-P_{12}P_{.1}(1-P_{1.}-P_{.2}) \\ -P_{11}P_{.2}(1-P_{1.}-P_{.1})-P_{12}P_{.1}(1-P_{1.}-P_{.2}), & P_{11}P_{.2}^{2}+P_{12}(1-P_{1.}-P_{.2})^{2}+P_{22}P_{1.}^{2}+P_{32}P_{1.}^{2} \end{matrix}\right] \\ -\left[\begin{matrix} P_{11}(1-P_{1.}-P_{.1})-P_{12}P_{.1}-P_{21}P_{1.}-P_{31}P_{1.} \\ -P_{11}P_{.2}+P_{12}(1-P_{1.}-P_{.2})-P_{22}P_{1.}-P_{32}P_{1.} \end{matrix}\right] \\ \left[P_{11}(1-P_{1.}-P_{.1})-P_{12}P_{.1}-P_{21}P_{1.}-P_{31}P_{1.},-P_{11}P_{.2}+P_{12}(1-P_{1.}-P_{.2})-P_{22}P_{1.}-P_{32}P_{1.}\right] \end{matrix}$$

(A1)

But, we can show

$\begin{matrix} P_{11}(1-P_{1.}-P_{.1})^{2}+P_{12}P_{.1}^{2}+P_{21}P_{1.}^{2}+P_{31}P_{1.}^{2} \\ =P_{11}(P_{.2}-P_{1.})^{2}+P_{12}P_{.1}^{2}+P_{21}P_{1.}^{2}+P_{31}P_{1.}^{2} \\ =(P_{11}+P_{21}+P_{31})P_{1.}^{2}+P_{11}P_{.2}^{2}-2P_{11}P_{1.}P_{.2}+P_{12}P_{.1}^{2} \\ =P_{1.}^{2}P_{.1}+P_{1.}P_{.1}P_{.2}^{2}-2P_{1.}^{2}P_{1.}P_{.2}+P_{1.}P_{.2}P_{.1}^{2} \\ =P_{1.}^{2}P_{.1}(1-P_{.2})+P_{1.}P_{.1}P_{.2}^{2}-P_{1.}^{2}P_{.1}P_{.2}+P_{1.}P_{.2}P_{.1}^{2} \\ =P_{1.}^{2}P_{.1}^{2}+P_{1.}P_{.1}P_{.2}(P_{.2}+P_{.1})-P_{1.}^{2}P_{.1}P_{.2} \\ =P_{1.}^{2}P_{.1}^{2}+P_{1.}P_{.1}P_{.2}-P_{1.}^{2}P_{.1}P_{.2} \end{matrix}$ (A2)

and

$\begin{matrix} -P_{11}P_{.2}(1-P_{1.}-P_{.1})-P_{12}P_{.1}(1-P_{1.}-P_{.2}) \\ =-(1-P_{1.})(P_{11}P_{.2}+P_{12}P_{.1})+P_{11}P_{.1}P_{.2}+P_{12}P_{.1}P_{.2} \\ =-(1-P_{1.})(P_{1.}P_{.1}P_{.2}+P_{1.}P_{.1}P_{.2})+P_{1.}P_{.1}^{2}P_{.2}+P_{1.}P_{.1}P_{.2}^{2} \\ =-2(1-P_{1.})P_{1.}P_{.1}P_{.2}+P_{1.}P_{.1}P_{.2} \\ =2P_{1.}^{2}P_{.1}P_{.2}-P_{1.}P_{.1}P_{.2} \end{matrix}$ (A3)

Similarly, we have

$P_{11}P_{.2}^{2}+P_{12}(1-P_{1.}-P_{.2})^{2}+P_{22}P_{1.}^{2}+P_{32}P_{1.}^{2}=P_{1.}^{2}P_{.2}^{2}+P_{1.}P_{.1}P_{.2}-P_{1.}^{2}P_{.1}P_{.2}$ (A4)

We notice that equations (A5) and (A6) also hold

$$\begin{matrix} P_{11}(1-P_{1.}-P_{.1})-P_{12}P_{.1}-P_{21}P_{1.}-P_{31}P_{1.}=P_{11}-(P_{11}+P_{21}+P_{31})P_{1.}-P_{11}P_{.1}-P_{12}P_{.1} \\ \begin{matrix} & =P_{11}-P_{1.}P_{.1}-P_{11}P_{.1}-P_{12}P_{.1}=P_{11}-P_{1.}P_{.1}-P_{1.}P_{.1}=-P_{1.}P_{.1} \end{matrix} \end{matrix}$$

(A5)

$\begin{matrix} -P_{11}P_{.2}+P_{12}(1-P_{1.}-P_{.2})-P_{22}P_{1.}-P_{32}P_{1.}=-P_{11}P_{.2}+P_{12}-(P_{12}+P_{22}+P_{32})P_{1.}-P_{12}P_{.2} \\ \begin{matrix} & =-P_{11}P_{.2}+P_{12}-P_{.2}P_{1.}-P_{12}P_{.2}=-(P_{11}+P_{12})P_{.2}=-P_{1.}P_{.2} \end{matrix} \end{matrix}$ (A6)

Combining equations (A1), (A2), (A3), (A4), (A5) and (A6) yields

$$\begin{matrix} \left[\begin{matrix} C_{1} \\ C_{2} \end{matrix}\right]\Sigma\left[\begin{matrix} C_{1}^{T} & C_{2}^{T} \end{matrix}\right]=\left[\begin{matrix} P_{1.}^{2}P_{.1}^{2}+P_{1.}P_{.1}P_{.2}-P_{1.}^{2}P_{.1}P_{.2} & 2P_{1.}^{2}P_{.1}P_{,2}-P_{1.}P_{.1}P_{.2} \\ 2P_{1.}^{2}P_{.1}P_{.2}-P_{1.}P_{.1}P_{.2} & P_{1.}^{2}P_{.2}^{2}+P_{1.}P_{.1}P_{.2}-P_{1.}^{2}P_{.1}P_{.2} \end{matrix}\right]-\left[\begin{matrix} P_{1.}^{2}P_{.1}^{2} & P_{1.}^{2}P_{.1}P_{.2} \\ P_{1.}^{2}P_{.1}P_{.2} & P_{1.}^{2}P_{.2}^{2} \end{matrix}\right] \\ \begin{matrix} & & & & & =\left[\begin{matrix} P_{1.}P_{.1}P_{.2}-P_{1.}^{2}P_{.1}P_{.2} & P_{1.}^{2}P_{.1}P_{.2}-P_{1.}P_{.1}P_{.2} \\ P_{1.}^{2}P_{.1}P_{.2}-P_{1.}P_{.1}P_{.2} & P_{1.}P_{.1}P_{.2}-P_{1.}^{2}P_{.1}P_{.2} \end{matrix}\right] \end{matrix} \end{matrix}$$

Thus, its determinant is equal to

$$\left|\left[\begin{matrix} C_{1} \\ C_{2} \end{matrix}\right]\Sigma\left[\begin{matrix} C_{1}^{T} & C_{2}^{T} \end{matrix}\right]\right|=(P_{1.}P_{.1}P_{.2}-P_{1.}^{2}P_{.1}P_{.2})^{2}-(P_{1.}P_{.1}P_{.2}-P_{1.}^{2}P_{.1}P_{.2})^{2}=0$$

Similarly, we can show that

$\left|\left[\begin{matrix} C_{3} \\ C_{4} \end{matrix}\right]\Sigma\left[\begin{matrix} C_{3}^{T} & C_{4}^{T} \end{matrix}\right]\right|=0$ and $\left|\left[\begin{matrix} C_{5} \\ C_{6} \end{matrix}\right]\Sigma\left[\begin{matrix} C_{5}^{T} & C_{6}^{T} \end{matrix}\right]\right|=0$

Next we calculate the determinant of the matrix $\left|\left[\begin{matrix} C_{1} \\ C_{3} \end{matrix}\right]\Sigma\left[\begin{matrix} C_{1}^{T} & C_{3}^{T} \end{matrix}\right]\right|$. By the similar argument, we have

$$\begin{matrix} \left[\begin{matrix} C_{1} \\ C_{3} \end{matrix}\right]\Sigma\left[\begin{matrix} C_{1}^{T} & C_{3}^{T} \end{matrix}\right]=\left[\begin{matrix} P_{1.}^{2}P_{.1}^{2}+P_{1.}P_{.1}P_{.2}-P_{1.}^{2}P_{.1}P_{.2} & 2P_{1.}P_{.1}^{2}P_{2.}-P_{1.}P_{.1}P_{2.} \\ 2P_{1.}P_{.1}^{2}P_{2.}-P_{1.}P_{.1}P_{2.} & P_{.1}^{2}P_{2.}^{2}+P_{2.}P_{.1}P_{.2}-P_{2.}^{2}P_{.1}P_{.2} \end{matrix}\right]-\left[\begin{matrix} P_{1.}^{2}P_{.1}^{2} & P_{1.}P_{2.}P_{.1}^{2} \\ P_{1.}P_{2.}P_{.1}^{2} & P_{2.}^{2}P_{.1}^{2} \end{matrix}\right] \\ \begin{matrix} & & & & & =\left[\begin{matrix} P_{1.}P_{.1}P_{.2}-P_{1.}^{2}P_{.1}P_{.2} & P_{1.}P_{.1}^{2}P_{2.}-P_{1.}P_{.1}P_{2.} \\ P_{1.}P_{.1}^{2}P_{2.}-P_{1.}P_{.1}P_{2.} & P_{2.}P_{.1}P_{.2}-P_{2.}^{2}P_{.1}P_{.2} \end{matrix}\right] \end{matrix} \end{matrix}$$

Thus, its determinant is equal to

$\left|\left[\begin{matrix} C_{1} \\ C_{3} \end{matrix}\right]\Sigma\left[\begin{matrix} C_{1}^{T} & C_{3}^{T} \end{matrix}\right]\right|=P_{1.}P_{2.}P_{.1}^{2}P_{.2}^{2}P_{3.}>0$, when $P_{1.}>0,P_{2.}>0,P_{3.}>0,P_{.1}>0,P_{.2}>0$

Next we show that $\left|\left[\begin{matrix} C_{1} \\ C_{3} \\ C_{5} \end{matrix}\right]\Sigma\left[\begin{matrix} C_{1}^{T} & C_{3}^{T} & C_{5}^{T} \end{matrix}\right]\right|=0$. In fact, we have

Thus, we obtain

$$\begin{matrix} \left|\left[\begin{matrix} C_{1} \\ C_{3} \\ C_{5} \end{matrix}\right]\Sigma\left[\begin{matrix} C_{1}^{T} & C_{3}^{T} & C_{5}^{T} \end{matrix}\right]\right|=P_{1.}P_{2.}P_{3.}P_{.1}^{3}\left|\begin{matrix} P_{.2}(1-P_{1.}) & P_{1.}(P_{.1}-1) & P_{1.}(P_{.1}-1) \\ P_{2.}(P_{.1}-1) & P_{.2}(1-P_{2.}) & P_{2.}(P_{.1}-1) \\ P_{3.}(P_{.1}-1) & P_{3.}(P_{.1}-1) & P_{.2}(1-P_{3.}) \end{matrix}\right| \\ =P_{1.}P_{2.}P_{3.}P_{.1}^{3}[(P_{.1}-1)^{2}P_{.2}(P_{1.}P_{2.}P_{3.}-P_{1.}P_{2.}-P_{1.}P_{3.}-P_{2.}P_{3.})+P_{.2}^{3}(1-P_{1.})(1-P_{2.})(1-P_{3.})] \\ =P_{1.}P_{2.}P_{3.}P_{.1}^{3}P_{.2}^{3}[P_{1.}P_{2.}P_{3.}-P_{1.}P_{2.}-P_{1.}P_{3.}-P_{2.}P_{3.}+(1-P_{1.})(1-P_{2.})(1-P_{3.})] \\ =P_{1.}P_{2.}P_{3.}P_{.1}^{3}P_{.2}^{3}(1-P_{1.}-P_{2.}-P_{3,}) \\ =0 \end{matrix}$$

Therefore, the rank of $C\Sigma C^{T}=2$. Similarly, we can show that the rank of $B\Sigma_{D}B^{T}=2$, which implies that the rank of $\Lambda$ under the null hypothesis of no gene-environment interaction is equal to 2.
