# Supplementary material for: Mutual Information for Testing Gene-Environment Interaction
Source: PLoS One. 2009 Feb 24;4(2):e4578. doi: 10.1371/journal.pone.0004578 (PMC2642626; doi:10.1371/journal.pone.0004578)
Supplement: Appendix S2 — (0.05 MB DOC) [file pone.0004578.s002.doc]

**APPENDIX S2**

Consider a candidate locus *G* with two alleles and an environmental exposure *E*. The genotypes at locus G with two, one and zero of risk increasing allele is coded as 2,1 and 0, respectively. Define the logistic regression model: $P(D=1|G,E)=\frac{e^{\alpha+\beta_{g}G+\beta_{e}E+\beta_{ge}GE}}{1+e^{\alpha+\beta_{g}G+\beta_{e}E+\beta_{ge}GE}}$.

By definition of the baseline probability of disease in the population, genetic, environment and genetic-environmental interaction odds ratio and the logistic regression model, we have

$$OR_{b}=\frac{P(D=1|G=0,E=0)}{P(D=0|G=0,E=0)}=e^{\alpha}$$

$$OR_{g}=\frac{\frac{P(D=1|G=1,E=0)}{P(D=0|G=1,E=0)}}{\frac{P(D=1|G=0,E=0)}{P(D=0|G=0,E=0)}}=e^{\beta_{g}}$$

$$OR_{e}=\frac{\frac{P(D=1|G=0,E=1)}{P(D=0|G=0,E=1)}}{\frac{P(D=1|G=0,E=0)}{P(D=0|G=0,E=0)}}=e^{\beta_{e}}$$

$$OR_{ge}=\frac{\frac{P(D=1|G=1,E=1)}{P(D=0|G=1,E=1)}}{OR_{b}OR_{g}OR_{e}}=e^{\beta_{ge}}$$

$$OR_{gg}=\frac{\frac{P(D=1|G=2,E=0)}{P(D=0|G=2,E=0)}}{\frac{P(D=1|G=0,E=0)}{P(D=0|G=0,E=0)}}=e^{2\beta_{g}}=OR_{g}^{2}$$

$$OR_{gge}=\frac{\frac{P(D=1|G=2,E=1)}{P(D=0|G=2,E=1)}}{OR_{b}OR_{gg}OR_{e}}=e^{2\beta_{ge}}=OR_{ge}^{2}$$

Solving the above equations, we obtain the following equations for calculations of the penetrance functions:

$$P(D=1|G=0,E=0)=\frac{OR_{b}}{1+OR_{b}}$$

$$P(D=1|G=1,E=0)=\frac{OR_{b}OR_{g}}{1+OR_{b}OR_{g}}$$

$$P(D=1|G=0,E=1)=\frac{OR_{b}OR_{e}}{1+OR_{b}OR_{e}}$$

$$P(D=1|G=1,E=1)=\frac{OR_{b}OR_{g}OR_{e}OR_{ge}}{1+OR_{b}OR_{g}OR_{e}OR_{ge}}$$

$$P(D=1|G=2,E=0)=\frac{OR_{b}OR_{gg}}{1+OR_{b}OR_{gg}}$$

$$P(D=1|G=2,E=1)=\frac{OR_{b}OR_{gg}OR_{e}OR_{gge}}{1+OR_{b}OR_{gg}OR_{e}OR_{gge}}$$

For calculation of the power of the test statistics in the text, we assume

$OR_{g}=1,OR_{e}=1,OR{}_{b}=0.01,OR_{gg}=OR_{g}^{2},OR_{gge}=OR_{ge}^{2}$.
